# EFFECTIVE STRATEGIES FOR PREPARING STRONG CAREER DEVELOPMENT AWARD (“K”) APPLICATIONS

**DOI:** 10.1093/geroni/igad104.0662

**Published:** 2023-12-21

**Authors:** Jamie Lahvic

**Affiliations:** NIH/National Institute on Aging, Bethesda, Maryland, United States

## Abstract

In this presentation, Dr. Lahvic will present critical policies, best practices, and resources to consider when preparing mentored career development award (“K”) applications. Dr. Lahvic will emphasize 1) Starting the process early; 2) Developing an application strategy, including reaching out to relevant program officials, identifying appropriate mentors and collaborators, and evaluating if the career development award will add value to your professional development trajectory; and 3) Understanding the application requirements. Using real, successful and unsuccessful career development applications as examples, Dr. Lahvic will highlight best practices in planning the research and career development strategy, including avoiding proposing an overly ambitious project and ensuring the project is a vehicle towards future independence and is distinct from mentor’s research. Knowledge of these practices and available resources to implement them will optimize the application process for individuals.

